# Supplementary material for: Localization, proteomics, and metabolite profiling reveal a putative vesicular transporter for UDP-glucose
Source: eLife. 2021 Jul 16;10:e65417. doi: 10.7554/eLife.65417 (PMC8373376; doi:10.7554/eLife.65417)
Supplement: Supplementary file 1. [file elife-65417-supp1.docx]

| **Family** | **Transporter** |
| --- | --- |
| Facilitative GLUT transporter family | SLC2A6 |
| Sodium glucose cotransporter family | SLC5A3 |
|  | SLC5A7 |
|  | SLC5A12 |
| Sodium- and chloride-dependent neurotransmitter transporter family | SLC6A7 |
|  | SLC6A14 |
|  | SLC6A17 |
| Cationic amino acid transporter/glycoprotein-associated family | SLC7A4 |
| Na^+^/H^+^ exchanger family | SLC9A1 |
| Sodium bile salt cotransport family | SLC10A4 |
| Folate/thiamine transporter family | SLC19A3 |
| Organic anion transporter family | SLC21A9 |
| Organic cation/anion/zwitterion transporter family | SLC22A6 |
|  | SLC22A24 |
| Mitochondrial carrier family | SLC25A13 |
|  | SLC25A40 |
| Multifunctional anion exchanger family | SLC26A11 |
| Zinc efflux family | SLC30A3 |
| Nucleoside-sugar transporter family | SLC35A2 |
|  | SLC35A3 |
|  | SLC35B1 |
|  | SLC35B4 |
|  | SLC35C1 |
|  | SLC35D2 |
|  | SLC35D3 |
|  | SLC35E4 |
|  | SLC35F1 |
|  | SLC35F2 |
|  | SLC35F3 |
|  | SLC35F6 |
|  | SLC35G2 |
| Proton-coupled amino acid transporter family | SLC36A3 |
| System A and System N sodium-coupled neutral amino acid transporter family | SLC38A4 |
|  | SLC38A6 |
| Metal ion transporter family | SLC39A5 |
| Basolateral iron transporter family | SLC40A1 |
| MgtE-like magnesium transporter family | SLC41A2 |
| Na^+^-independent, system-L-like amino acid transporter family | SLC43A3 |
| Heme transporter family | SLC48A1 |
| Riboflavin transporter family | SLC52A3 |

**Supplementary File 1. Vesicular transporters identified in SLC localization profiling (related to Figure 1)**
