# Supplementary material for: Localization, proteomics, and metabolite profiling reveal a putative vesicular transporter for UDP-glucose
Source: eLife. 2021 Jul 16;10:e65417. doi: 10.7554/eLife.65417 (PMC8373376; doi:10.7554/eLife.65417)
Supplement: Supplementary file 2. [file elife-65417-supp2.docx]

| **Family** | **Transporter** | **Reported** | **Ref** |
| --- | --- | --- | --- |
| Vesicular glutamate transporter | SLC17A6 (VGLUT2) | Yes | Known vesicular transporters |
|  | SLC17A7 (VGLUT1) | Yes |  |
|  | SLC17A8 (VGLUT3) | Yes |  |
| Vesicular amine transporter | SLC18A2 (VMAT2) | Yes |  |
|  | SLC18A3 (VAChT) | Yes |  |
| Vesicular inhibitory amino acid transporter | SLC32A1 (VGAT) | Yes |  |
| Zinc efflux family | SLC30A3 (ZNT3) | Yes | (Palmiter et al., 1996) |
| Facilitative GLUT transporter | SLC2A3 | Yes | (Gronborg et al., 2010) |
|  | SLC2A13 | Yes | (Takamori et al., 2006) |
| Cationic amino acid transporter/glycoprotein-associated | SLC7A14 | Yes | (Takamori et al., 2006) |
| Na^+^/Ca^2+^ exchanger | SLC8A1 | Yes | (Gronborg et al., 2010) |
|  | SLC8A2 | Yes | (Gronborg et al., 2010) |
| Na^+^/H^+^ exchanger | SLC9A7 | Transport activity reported | (Goh et al., 2011) |
| Sodium- and chloride-dependent neurotransmitter transporter | SLC6A7 | Yes | (Renick et al., 1999) |
|  | SLC6A17 | Yes | (Parra et al., 2008)  (Zaia and Reimer, 2009). |
|  | SLC6A1 | No |  |
| Heavy subunits of the heteromeric amino acid transporter | SLC3A2 | No |  |
| Bicarbonate transporter | SLC4A10 | No |  |
| Sodium glucose cotransporter | SLC5A7 | No |  |
| Electroneutral cation-coupled Cl cotransporter | SLC12A7 | No |  |
|  | SLC12A6 | No |  |
|  | SLC12A9 | No |  |
| Type III Na^+^-phosphate cotransporter | SLC20A2 | No |  |
| Organic cation/anion/zwitterion transporter | SLC22A17 | No |  |
| Nucleoside-sugar transporter | SLC35D3 | No |  |
|  | SLC35F1 | No |  |
|  | SLC35G2 | No |  |

**Supplementary File 2. SLC transporters enriched in immunoisolated synaptic vesicles (related to Figure 2)**
